# Radiotherapy for High-Grade Gliomas in Adults and Children: A Systematic Review of Advances Published in the Second Half of 2023

**DOI:** 10.3390/ijms27020662

**Published:** 2026-01-09

**Authors:** Guido Frosina

**Affiliations:** Neuro-Oncology & Mutagenesis Unit, IRCCS Ospedale Policlinico San Martino, Largo Rosanna Benzi 10, 16132 Genova, Italy; guido.frosina@hsanmartino.it or guidofrosina7@gmail.com; Tel.: +39-010-5558543; Fax: +39-010-5558237

**Keywords:** clinical, fractionation, planning, malignant brain tumor, preclinical, radiosensitisation, adult, pediatric

## Abstract

While research on high-incidence tumors such as breast, prostate, and lung cancer has led to significant increases in patient survival in recent years, this has not been the case for low-incidence tumors such as high-grade gliomas, the most common and lethal brain tumors, for which the last significant therapeutic advance dates back to 2005. The high infiltration capacity of these tumors into normal brain tissue essential for both vegetative and relational life, the tumor microenvironment, with poor immunological activity, the multiple resistance mechanisms, and the unattractiveness of research investments due to the limited number of patients have made, and continue to make, the path to achieving significant improvements in the survival of patients with high-grade gliomas long and arduous. The objective of this article is to update the slow but continuous radiotherapeutic progress for adult and pediatric high-grade gliomas to the second half of 2023. We analyzed the progress of preclinical and clinical research on both adult and pediatric high-grade gliomas, with a particular focus on improvements in radiotherapy. Interactions between non-radiant new therapies and radiotherapy were also covered. A literature search was conducted in PubMed using the terms (“glioma* and radio*”) and the time limit of 1 July 2023 to 31 December 2023. The inclusion and exclusion criteria for the review were relevance to advances in radiotherapy for high-grade gliomas in adults and children. Treating patients with advanced disease progression only, using “historical” data as controls, as well as repurposing drugs developed for purposes completely different from their intended use, were the major (but not the only) methods to assess risk of bias in the included studies. The effect measures used in the synthesis or presentation of the results were tabulated and/or displayed in figures. A total of 100 relevant references were reviewed. Advances in preclinical studies and in clinical radiotherapy treatment planning, innovative fractionation, use of radioisotopes/radiopharmaceuticals, radiosensitization procedures, and radiation-induced damage were focused on. While this analysis may be limited by the relatively short publication period, high-grade glioma research remains impacted, especially at the clinical level, by potential issues with trial design, such as treating patients with advanced disease progression, using “historical” data as controls, and repurposing drugs developed for completely different purposes than intended. Addressing these aspects of high-grade glioma research could improve its efficacy, which often remains low despite the associated costs.

## 1. Introduction

High-grade gliomas (HGG) are characterized by infiltrative capacity and resistance to therapies. Traditionally, they were diagnosed via histology, but these diagnoses left large differences in the reporting by different pathologists and a vast heterogeneity of prognosis between patients, even within the same histopathological grade [1]. Gradually, the histological diagnosis was supplemented by analyses of biomolecular markers, which enabled an increase in diagnostic resolution and a narrowing of the areas of reporting variability [2]. This combination of histology and biomolecular analysis has continued to evolve in subsequent editions of the classification of brain tumors, which reached its fifth edition in 2021. Adult HGGs are currently divided into four groups: (1) grade III oligodendroglioma [1p/19 co-deleted, isocitrate dehydrogenase (*IDH*)-mutant]; (2) grade III *IDH*-mutant astrocytoma; (3) grade IV IDH-mutant astrocytoma; (4) grade IV *IDH* wild-type glioblastoma (GB) [3]. Pediatric HGGs are currently divided into four subtypes: (1) Diffuse midline glioma (DMG) with *H3K27* mutation. Midline involvement encompasses the pons, as well as other key locations, including the thalamus, hypothalamus, pineal region, cerebellum, and spinal cord. The prognosis is generally poor. (2) Diffuse hemispheric glioma with *H3G34* mutation. The prognosis is usually poor. (3) Pediatric high-grade diffuse glioma, *H3*-wildtype and *IDH*-wildtype. Often has *MYCN* amplification, *PDGFRA* amplification, and/or *TERT* mutation, with the former (*MYCN* amplification) associated with shorter overall survival (OS). (4) Infantile-type hemispheric glioma: the most evident difference compared to the previous ones is the age of the patients, which is limited to childhood [3]. Typical genetic alterations in HGGs further involve the tyrosine kinase genes *ROS1*, *ALK*, *MET*, and the *NTRK* family. It is easy to foresee that, despite the above details, future advances in molecular, morphological, and clinical aspects for both adult and pediatric HGGs, as well as knowledge of genetic alterations, age distribution, variability of tumor location, variability of histopathological and radiological findings, prognosis, and outcome, will lead to increasingly complex classifications in the coming years. To present a couple of cases as examples, Hadad et al. identified a subgroup of treatment-naïve *IDH*-wildtype primary GBs in adults (2%, 9/459), characterized by somatic hypermutation and defective mismatch repair (MMR), and named it ‘de novo replication repair deficient GB, *IDH*-wildtype [4]. Tantillo and co-workers further suggested that some HGGs can induce changes in pyramidal neurons that drive further tumor progression [5].

The standard treatment for HGG includes, in addition to surgery, radiotherapy (RT) and chemotherapy (CT), mainly with temozolomide (TMZ). This combination can guarantee patient survival of more than 10 years for some *IDH1/2* mutant grade III gliomas. For grade IV tumors, median survival typically does not exceed a year and a half. Molecular profiling is still exploited for therapeutic purposes for a small minority of patients, such as those affected by an HGG with *BRAF* (V600E) mutations (i.e., Val600Glu) [6]. The great geno/phenotypic heterogeneity of HGGs constitutes one of the major obstacles to improving their treatment, with a stalemate dating back twenty years [7]. Here, we discuss the main advances in HGG therapy published during the second half of 2023, with a particular focus on RT treatments. Interactions between non-radiant new therapeutic strategies and RT will also be covered.

## 2. Materials and Methods

The PRISMA guidelines were followed. Briefly, a literature search was conducted using the terms (“gliom* and radio*”) and the time limit 1 July 2023 to 31 December 2023. PubMed software (the National Center for Biotechnology Information (NCBI), at the U.S. National Library of Medicine (NLM), located at the National Institutes of Health (NIH). Bethesda, MD, USA. https://pubmed.ncbi.nlm.nih.gov/) was searched or consulted to identify studies and was last accessed on 30 December 2025. While the use of a single database search may limit the retrieval of contributions, we believe this may be acceptable for a focused 6-month update. The inclusion and exclusion criteria for the review were relevance to advances in RT for HGG in adults and children. Out of 730 bibliographic records retrieved automatically, 215 were deemed relevant to the topics “RT of HGG” or “HGG therapies other than RT” by the author based on their titles. Of these, 214 were confirmed to be eligible based on the abstracts. All 214 full-text PDF files were retrieved. Eighty-three of these were found to be specifically relevant to the “RT of HGG” topic and are discussed in this review. An additional seventeen relevant records published outside the second half of 2023 contribute to the discussion, bringing the total to 100 references. A total of 131 references published in the second half of 2023 concerning “HGG therapies other than RT” will be discussed elsewhere.

**Figure 1:**
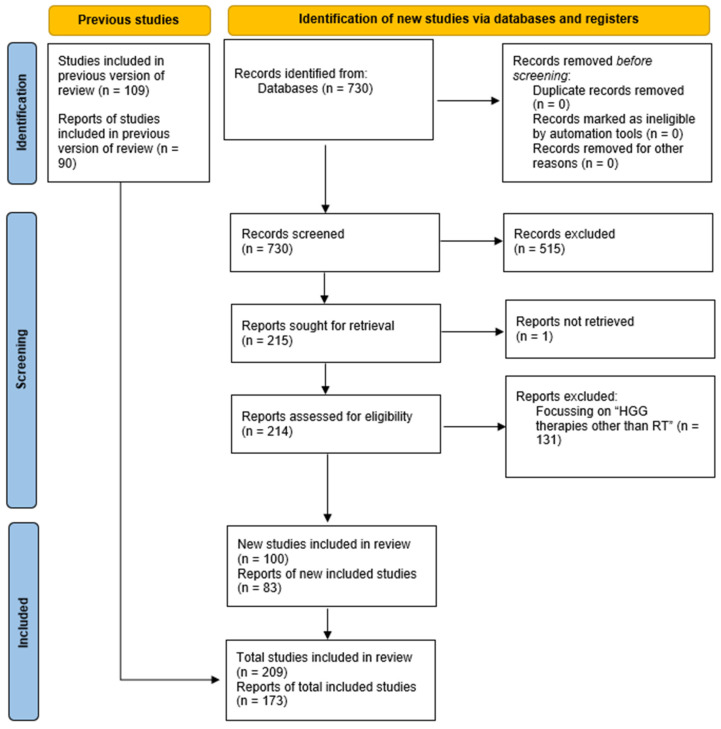
Flow diagram of reported studies [modified from refs [7,8], with permission].

Studies were grouped for synthesis based on the titles of their sections and subsections. Treating patients with advanced disease progression only, using “historical” data as controls, as well as repurposing drugs developed for purposes completely different from their intended use, were major (not exhaustive) methods to assess risk of bias in the included studies. The effect measures (risk ratio, mean difference) used in the synthesis or presentation of the results are reported in the Tables and Figures and in the original articles. Tabulating the study intervention characteristics and comparing them against the planned groups were the processes used to decide which studies were eligible. The selection of the most significant results, especially relevant to survival improvement, was the method to prepare the data for presentation or synthesis. The results of individual studies and syntheses were tabulated or visually displayed in Tables and Figures, respectively. The main conclusions of each study were compared to the study authors’ conclusions and used to synthesize the results. Subgroup analysis was used to explore possible causes of heterogeneity among study results. Statistical analyses were used to assess the robustness of the reported results as well as confidence in the body of evidence for an outcome. The relevant PRISMA 2020 flowchart and checklists are presented in Figure 1, Figures S1 and S2, respectively [8].

## 3. Results and Discussion

### 3.1. Radiotherapeutic Progress for HGG

#### 3.1.1. Adult HGG

##### Preclinical Studies

Several promising preclinical studies on the radiotherapeutic (RT) progress for HGGs were published during the second half of 2023. The stage from translational development to clinical utility is variable, and some topics have uncertain translational timelines. More deeply studied and advanced approaches reporting possible clinical trial-worthy strategies are indicated with “*” in Table 1.

**Figure 2:**
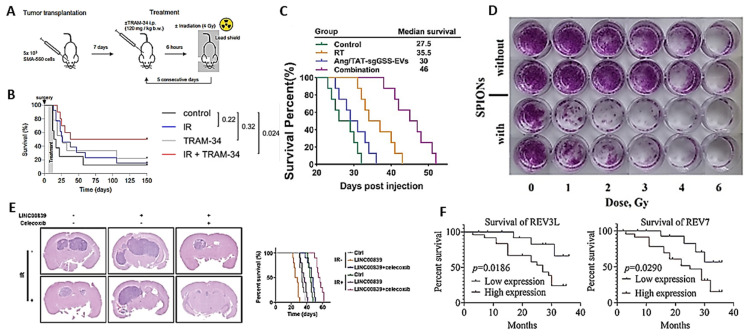
Preclinical studies. (**A**,**B**) Combined irradiation and TRAM-34 therapy increases survival in the SMA-560 VM/Dk glioma model. (**A**) Schematic of tumor transplantation and subsequent treatment. (**B**) The Kaplan–Meier estimator started on the day of tumor cell injection into the right striatum. Arrowhead depicts the day of surgery; the treatment period is shaded in grey. Animals in the control group are shown in black, animals in the irradiation group in blue, animals in the TRAM-34-only group in grey, and animals in the combined irradiation + TRAM 34 group in red. Numbers in (**B**) indicate *p* values, as calculated by the log-rank Mantel–Cox test (after [9], with permission). (**C**) Therapeutic effect of Ang/TAT-sgGSS-EVs on patient-derived GIC xenografts. Survival curves of LN229-bearing mice (after [12], with permission). (**D**) Comparison of cell survival under combined exposure to 100 µg (Fe)/mL of SPIONs and X-ray radiation at different absorbed doses. Examples of plate photographs of the GB Gl-Tr cells, visualized using crystal violet (after [13], with permission). (**E**) Wnt/β-catenin inhibitor inhibits GB growth and sensitizes GICs to IR. **Left**. Representative images of hematoxylin and eosin-stained cross-sections of tumor-bearing brains after radiation and celecoxib treatment. **Right**. Kaplan–Meier survival curves of immunocompromised mice bearing intracranially injected GICs after radiation and celecoxib treatment (after [14], with permission). (**F**) The prognosis of patients with glioma is influenced by the high or low (left) REV3L and (right) REV7 expression. The different expressions of REV3L and Rev7 in tissues were assessed using Student’s *t*-test. The OS of patients with glioma was evaluated using Kaplan–Meier analysis (after [18], with permission).

Stransky et al. investigated the intermediate conductance calcium-activated potassium channel K(Ca)3.1 as a novel therapeutic target for HGGs (Table 1, Figure 2A,B) [9]. Using the syngeneic and immunocompetent orthotopic mouse model SMA-560/VM/Dk, they observed that the inhibition of the calcium channel *via* the specific drug TRAM-34 combined with RT caused radiosensitization of the tumor and increased the survival of the mice in the absence of effects on the T-cell-mediated immune response.

Using a linear accelerator, Yan, Yu, and colleagues studied the effect of Wnt/β-catenin inhibitors on the efficacy of RT at low (0.3 Gy) or high (3.0 Gy) doses towards C6 HGG (Table 1) [10]. While low doses caused increased proliferation and migration of C6 glioma cells, 3.0 Gy doses inhibited these phenomena. Their combination with the Wnt1 inhibitor IWR1 resulted in a significant reduction in the proliferation, migration, and expression of Wnt1, Wnt3a, and β-catenin proteins in C6 cells, as well as their radiosensitization.

Ferroptosis is a type of programmed cell death that depends on the excessive accumulation of free iron within brain cells. This alteration in iron homeostasis in turn causes lipid peroxidation phenomena, which damage brain cells. Parenthetically, the production of peroxides is a well-known mechanism of many anticancer agents that, unfortunately, in addition to being cytotoxic, are also mutagenic, increasing the risk of tissue degeneration and secondary carcinogenesis [64]. Ferroptosis can be the basis of various brain pathologies, including neurodegenerative diseases such as Parkinson’s and Alzheimer’s diseases, strokes, and tumors such as glial tumors. SOAT1 is a gene that has been investigated for its possible involvement in ferroptosis phenomena (Table 1) [11]. Its mechanism of action is unclear, but its impact on the prognosis of HGG patients has been recognized. The inhibition of SOAT1 increases the efficacy of ionizing radiation on HGG cells both in vitro and in vivo, as well as their sensitivity to ferroptosis.

The use of CRISPR/CAS genome editing technology in new therapeutic strategies for brain diseases has been discussed by Forgham and collaborators [65]. Liu, X. et al. carried out an investigation using CRISPR to identify genes that may affect the cytotoxicity of RT in orthotopic gliomas subjected to treatment with ionizing radiation (Table 1, Figure 2C) [12]. They identified glutathione synthetase (GSS) as a regulatory element of the response to RT. Elevated GSS levels correlate with a poorer prognosis for glioma patients. From a mechanistic point of view, GSS after irradiation would cause a lower induction of ferroptosis and a lower efficacy of RT. The depletion of GSS and consequent interruption of glutathione synthesis would allow for the accumulation of iron and reactivation of ferroptosis, which are capable of mediating the cytotoxicity of RT.

Nanotechnology techniques include different treatments, such as photodynamic therapy (PDT), photothermal therapy (PTT), chemodynamic therapy (CDT), sonodynamic therapy (SDT), and RT [66]. One type of these nanoparticles was engineered with a dextran-coated iron oxide core and tested on GB A172 and Gl-Tr cells in vitro (Table 1, Figure 2D) [13]. The nanoparticles absorbed by the glioma cells may have had a radiosensitizing action thanks to the emission of electrons from the iron oxide nucleus. The dextran shell was relatively stable throughout the procedure.

Long non-coding RNAs can serve as tumor biomarkers in certain cases [67]. The oncogenic function of LINCs has been studied in glioma-initiating cells (GICs), which are custodians of the HGG tumor’s initiation, maintenance, and therapeutic resistance. In GICs, LINC00839 was found to be overexpressed and appeared to exert the oncogenic and RT resistance effect by activating Wnt/β-catenin signaling (Table 1; Figure 2E) [14].

Derby and colleagues confirmed and extended previous studies suggesting that inhibition of the ataxia telangiectasia kinase Rad-related protein 3 (ATR) could lead to radiosensitization of HGGs (Table 1) [15]. This phenomenon could also be mediated by the destruction of the cytoskeletal structure and the consequent formation of intracytoplasmic integrins. This new mechanism of the radiosensitizing action of the pharmacological inhibition of ATR was studied both in vitro and in orthotopic animal models in vivo.

Protons (H^+^) and molecular hydrogen (H_2_) interact in the cell and are essential in a wide variety of processes. It has been suggested that H_2_ may have therapeutic effects on HGG diseases. H_2_ has antioxidant, anti-inflammatory, and antiapoptotic effects, and its inhalation could have some therapeutic effect on intracranial HGG treatment in orthotopic animal models (Table 1) [16]. The ability of GICs to form spheres was decreased after exposure to H_2_, as well as migration and colony formation. Although probably not linked to anti-inflammatory effects, but rather to physical properties of the type of radiation, proton RT may ultimately have more intense and localized therapeutic effects than photon RT when used in combination with H_2_.

In addition to targeting specific genetic factors in HGG induction and progression, intervening in more general metabolic processes might also be beneficial (Table 1). In HGG, glucose metabolism is reprogrammed, and it has been suggested to target genes involved in this reprogramming, such as PKM2, to enhance the effectiveness of RT [17].

The hedgehog (HH) signaling pathway and glioma-associated oncogene (GLI) are abnormally activated in several tumors and are believed to be associated with various mechanisms involved in tumor progression. Inhibitors of these oncogenic activities could therefore improve existing therapies for various types of cancer, including gliomas, but are currently not routinely used in the clinic due to their serious side effects [68]. DNA polymerase ζ may be another factor influencing the efficacy of RT in gliomas. The silencing of the genes that encode it (REV3L and REV7) causes inactivation of the PI3K/AKT/mTOR biochemical pathway, which in turn leads to decreased tumor resistance to RT (Table 1; Figure 2F) [18]. The ζ polymerase might be considered as a resistance factor for which inhibitors could be developed.

In addition to being used for the fluorescence analysis of HGGs during surgical removal to better identify the tumor tissues to be removed, 5-aminolevulinic acid (5-ALA) could also have a radiosensitizing effect. Suzuki and collaborators explored this possible effect on lymphoma cells cultured in vitro (Table 1) [19]. In flow cytometric analysis, 5-ALA-treated lymphoma cells exhibited 5-ALA-induced protoporphyrin (PpIX) accumulation and reduced survival upon irradiation, compared to control cells not treated with 5-ALA under both normal and hypoxic conditions.

##### Clinical Studies

*RT Treatment Planning*

**Figure 3:**
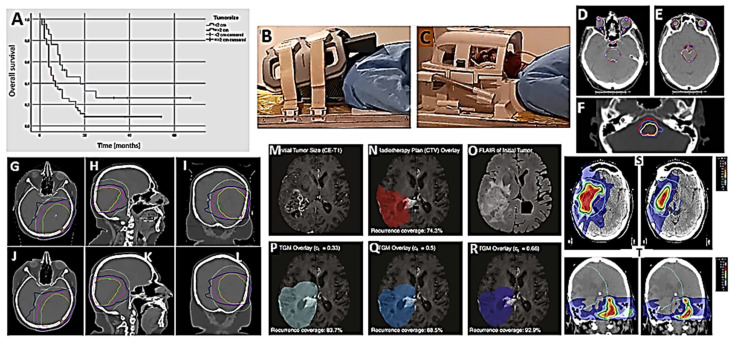
Clinical studies. Advances in RT for adult HGG patients. (**A**) Kaplan–Meier graph of overall survival (OS) of high-grade glioma (HGG) patients treated with SRT according to tumor size (after [20], with permission). (**B**) RT setup by using fixing devices (thermoplastic mask, fixing base, and headrest) and six-channel body matrix coil; (**C**) diagnostic setup (DS) with a head coil (after [21], with permission). (**D**–**F**) CTOM axial scans showing examples of the predicted CTOM deep learning segmentations compared to the gold-standard segmentation of the orbits (**D**,**E**), lenses (**D**), optic nerves (**D**), brainstem (**D**–**F**), optic chiasm (**E**), cochlea (**F**), lacrimal glands (**D**,**E**), and pituitary (**D**). Red represents the gold-standard segmentation. Clinical contours edited based on CTOM anatomy (CCeCTOM) are shown in yellow, while original clinical contours [CT unedited autosegmentation (CTu)] are in blue (after [22], with permission). (**G**–**L**) A case example of CTOM (**G**–**I**) versus deep-learning-based synthetic computed tomography (sCTOM) (**J**–**L**) image quality in a glioma patient with identical windowing parameters, showing the PTV (red) and the 2 cm normal tissue (NT) (light green) structure outlines. Relative isodose contours of 95% (green), 70% (magenta), and 50% (blue) are visible (after [24], with permission). (**M**–**R**) Comparison of standard clinical target volume (CTV) and computed target delineations derived from isolines of different estimated tumor cell densities by a tumor growth model (TGM). Underlying images are contrast-enhanced T1 (CE-T1). Exemplary case of a patient with recurrent GB. (**M**) Depicts the initial size of the tumor in CE-T1 imaging. (**N**) Shows the CTV planned on postoperative imaging and registered to the recurrence image that is visible underneath the overlay (**O**). Subparts (**P**–**R**) illustrate target volumes derived from isolines of estimated tumor cell density by the growth model (TGM) with cutoff values of ct = 0.33 (**P**), ct = 0.5 (**Q**), and ct = 0.66 (**R**). Note that the percentage of recurrence coverage improves with a lower cutoff value, and is, in this case, worst with traditional RT planning. However, there was also an increase in tumor volume with a relative volume ratio of 1.03, 1.25, and 1.52. Therefore, a reasonable trade-off needs to be found between improved recurrence coverage and as small a radiation volume as possible (after [26], with permission). (**S**) Patient 5 and (**T**) patient 10 both underwent the STRIDeR approach (**left**) and manual approach (**right**). ReRT PTV contour is represented by an orange outline, previous RT PTV contour is represented by a light blue outline, and brainstem contour is represented by a royal blue outline. Greater compromise is observed in the manual plan as a result of the maximum dose received by the brainstem PRV, used to determine the dose remaining for the brainstem superior, mid, and inferior divisions. In contrast, the STRIDeR approach allows for voxel-by-voxel EQD2 optimization, enabling the appropriate local placement of the dose in the brainstem, resulting in improved PTV coverage (after [27], with permission).

Soykut and colleagues studied the factors that influence the effectiveness of RT, in their case, stereotaxic (Table 1; Figure 3A) [20]. Of course, the therapeutic effect was significant, especially in tumors less than 2 cm in diameter. They suggested treating the lesions as early as possible, and in case of recurrence, treating that too, while they are still small in diameter.

MRI is preferred for tumor diagnostics, while computed tomography (CTOM) is primarily used for simulation. Rostani and collaborators studied the effect of patient immobilization devices during MRI and RT [21] (Figure 3B,C). The reduction in MRI quality was dependent on the brain region analyzed and its position relative to the coil. Despite the worsening of the images, the latter were still sufficient for an adequate diagnosis of the tumor and its response to treatments. The loss of resolution was certainly less than that which can occur with a patient who cannot sit still.

Contouring organs at risk (OAR) based on brain magnetic resonance imaging (MRI) is essential to reduce organ damage during RT. Alzahrani and collaborators trained and evaluated CTOM and MRI OAR auto-segmentation models (Table 1; Figure 3D–F) [22]. The procedure was found to be able to operate on all OARs, except the lacrimal glands. The effectiveness of the auto-segmentation model was highly dependent on the contour set.

Irannejad and collaborators attempted to reduce RT treatment planning time by utilizing deep learning for determining 3D dose distributions. Comparative analyses revealed no significant differences when including or excluding OARs from target volume (PTV) planning (Table 1) [23]. They concluded that OARs can be excluded from treatment planning with no particular consequences for accuracy but significant time savings. All this awaits confirmation in independent studies with a large number of patients.

Ranta et al. reported an RT treatment planning method using only MRI data for calculating the dose to be delivered and defining patient positioning (Table 1; Figure 3G–L) [24].

Häger et al. developed a mathematical model to describe how tumor cells infiltrate normal parenchyma, delineate the critical target, and possibly increase patients’ chances of survival (Table 1) [25]. This model predicted the OS of patients to be better than segmented gross tumor volume (GTV), suggesting that models predicting infiltration of normal parenchyma may confer a clinical advantage in RT treatment planning.

Metz et al. developed an experimental model of tumor growth that was more consistent with the clinical findings of tumor growth. The improved fidelity and reproducibility of experimental models of tumor growth patterns in the patient could provide advantages in treatment planning (Table 1; Figure 3M–R) [26].

Several studies suggest that a second course of irradiation, with or without other therapies, may be of benefit to patients in relapse. However, this remains a highly controversial topic for which we do not yet have clear limits and reproducibility conditions [69]. The STRIDeR (Support Tool for Re-Irradiation Decisions Guided by Radiobiology) planning pathway is intended to guide appropriate and biologically meaningful re-irradiation procedures. Thompson et al. compared the results of RT planning performed with STRIDeR and RT planning performed manually. They suggest that a combination of manual planning supported by STRIDeR could provide the best planning results with improved PTV doses (Table 1; Figure 3S,T) [27].

The prognosis of fourth-degree glioma is better if the IDH1/2 genes are mutated. In addition to having increased OS and PFS compared to patients with wild-type IDH, patients with mutated IDH are more radiosensitive. Han et al. investigated the possible mechanism by which the IDH mutation influences sensitivity to RT (Table 1; Figure S3) [28]. After analysis of 83 patients with fourth-degree glioma and the Cancer Genome Atlas (TCGA) and the Chinese Glioma Genome Atlas (CGGA) databases, they identified four additional genes, ADD3, GRHPR, RHBDL1, and SLC9A9, that may influence the efficacy of RT. If not subjected to RT, patients with this genetic profile did not show significant changes in survival.

*Innovative Fractionation*

Stereotactic radiosurgery (SRS) allows the radiation treatment to be tailored to the tumor, largely sparing the surrounding healthy tissues. It has proven effective against primary malignant and benign tumors, as well as secondary tumors (metastases). Three phase III studies are underway to determine the efficacy and safety of SRS against brain metastases [70]. SRS is usually carried out using photons, but it is also possible to use high-LET particle radiation, which has the advantage of delivering greater therapeutic energy to the tumor target and reducing involvement of the surrounding normal tissues. Mantica et al. performed a phase II study of the effect of SRS on the peripheral zone of the tumor, in combination with bevacizumab (BV), in patients facing relapse (Table S1) [71]. This treatment was well tolerated and could result in a temporary improvement in quality of life and PFS. No change in OS was recorded.

Re-irradiation with hyperfractionated RT with low and repeated doses has proven effective in the treatment of some ENT tumors, such as those of the nasopharynx [72]. Prospective randomized studies, such as the European Society for Radiotherapy and Oncology and European Organisation for Research and Treatment of Cancer (ESTRO-EORTC) observational cohort ReCare (NCT: NCT03818503), may provide information about the possible efficacy and toxicity of reirradiation against other neoplastic forms, including HGG.

Very elderly and frail HGG patients are often prescribed hypofractionated RT with 10–15 doses greater than 2 Gy. Kim et al. reported that increasing the overall dose using 20 fractions could have a significant effect on patient survival, without any particular drawbacks (Table 1) [29].

Baviskar et al. studied the effects of hypofractionated RT on HGG patients with poor prognosis (Table 1) [30]. The quality of life was temporarily increased, indicating that this RT regimen can represent an important palliative treatment that is relatively bearable and not economically burdensome.

Gregucci et al. evaluated the efficacy of SRS or fractionated stereotactic RT (FSRT) in association with regorafenib (Table 1) [31]. The combined treatment was found to be well tolerated; however, more numerous and randomized studies are needed to assess its effectiveness.

Marwah et al. discussed the effectiveness of re-irradiation techniques alone or in combination with chemotherapy (CT), as shown in Reference [73]. A combination of re-irradiation and BV may provide some benefit, especially in terms of decreased oedema and radionecrosis and improved PFS, while changes in OS remain insignificant. Intraoperative RT (IORT) enables the irradiation of residual disease in the operating cavity with single, effective high doses, thereby partially sparing the surrounding healthy tissues. This could therefore have an even more important role in effectively treating malignant brain tumors, which typically regrow at the site of the surgically removed tumor. We have less experience in the IORT of brain metastases, which is often found in the posterior cranial fossa and which requires more study from the point of view of both the surgical removal technique and of the possibly subsequent IORT. Krauss and collaborators reported a relatively safe surgery plus IORT technique for the removal of metastases in the posterior cranial fossa of nine patients (Table 1) [32].

Tumors of the pineal region are extremely rare and are characterized by great heterogeneity. They are typically treated with gamma knife radiosurgery (GKSR). This modality is the first choice in the case of primary tumor and also during recurrence. Gagliardi and collaborators discussed this topic and concluded the relative efficacy and safety of GKSR treatment for tumors of the pineal region [74].

A large body of preclinical studies continues to demonstrate that additional low doses of radiation [ultra-hyperfractionated radiotherapy (UHFRT)] can potentiate the effects of conventional RT and CT on cancer [7,72]. UHFRT illustrates the discontinuity of the dose–response curve and presents the counterintuitive observation that many small doses can produce an overall therapeutic effect that is superior to a single total dose [75]. Various mechanisms may explain this potentiation, including the progressively toxic (but incapable of triggering a repair response) accumulation of microamounts of DNA damage in tumor cells and the remodeling of the immune system [76]. Despite this plethora of preclinical evidence, the phenomenon has been poorly studied in HGG patients (e.g., limited to the treatment of patients with already-progressed tumors), and randomized, controlled trials in patients with minimal residual disease (e.g., those who recently completed the Stupp protocol) are needed [77].

*Radioisotopes and Radiopharmaceuticals*

The introduction of new radioisotopes with high LET (linear energy transfer) and low half-life can allow for the effective treatment of tumors without serious damage to normal tissues. In particular, actinium-225 (^225^Ac) emits heavy and energetic alpha particles with a high cytotoxic capacity [78]. Various radiopharmaceuticals have been developed for therapeutic purposes, exploiting the cytotoxic action of the particles emitted. Among them are ^225^Ac-PSMA-617, ^225^Ac-DOTATOC, and ^225^Ac-DOTA-substance-P, which have been used in patients suffering from various neoplasms, including gliomas. However, quality control techniques such as radiochemical purity require improvement.

Liang, R. et al. investigated whether it is possible to combine the techniques of nuclear medicine imaging and radiology to analyze the therapeutic capacity of a large panel of experimental radiopharmaceutical nanomedicines for HGG (Table 1) [33]. This multimodal imaging-guided strategy permitted the development of a lutetium-177 (^177^Lu)-labelled metal–organic nanomedicine.

Boron neutron capture therapy (BNCT) has been studied for a long time for the treatment of HGG. However, borated compounds often have a poor ability to pass the BBB and specifically target the tumor tissue. The topic has been discussed by Lan and coworkers [79]. A possible alternative to the most commonly used drug at present, namely 4-borono-L-phenylalanine (L-BPA), was proposed by Fujikawa and collaborators (Table S1) [80]. Boron-conjugated 4-iodophenylbutanamide (BC-IP) has been reported to have a good ability to cross the BBB and deliver boron to the tumor site. The characteristics of the two drugs were compared.

*Radiosensitization*

Krauze et al. performed a study to evaluate the changes in the proteomic profile in GB patients undergoing standard RT/CT treatment with or without valproic acid (VA) treatment (Table 1; Figure S3) [34]. The protein profile effectively changed after treatment with VA and showed the induction of protein markers typical of this drug. New markers with predictive potential regarding tumor response to VA were also identified.

Yuan and colleagues reported that a combination of TMZ and IMRT could synergistically improve the serum factor level in patients with postoperative HGG, enhance their immune function, and improve their clinical response (Table 1) [35]. The combination of TMZ with BV in glioma grade II and III patients who were treated with RT and relapsed has been investigated in an international, randomized, controlled phase II study (Table 1) [81]. No significant differences in OS were observed, nor were significant differences in patients’ quality of life and other secondary endpoints detected. However, Annakib and collaborators reported a limited beneficial effect of BV administration on the long-term quality of life of HGG patients who had undergone previous courses of RT and CT [36]. Differences in patient selection, timing, and/or combination regimens may have contributed to this variability in outcomes. The benefit of BV treatment in patients with HGG remains unclear and is likely dependent on factors that are not yet sufficiently defined. These factors may include specific genetic profiles that improve patient response to bevacizumab.

The methylation status of the promoter of the DNA repair enzyme O^6^-methylguanine methyltransferase (MGMT) correlates with a better prognosis for HGG patients, for reasons that are not yet fully understood. Buyuktepe et al. found that the promoter was methylated in 38.2% of cases and highly methylated in 10.5% of cases (Table 1) [37]. The CpG islands in the promoter region that were most closely associated with increased patient survival were CpG 79–83 and CpG 84–87. The presence of the mutated IDH gene next to the methylated MGMT promoter leads to a further increase in the progression-free survival (PFS) of patients. The beneficial effect of this genetic profile was found by Zhong and colleagues in a retrospective study of HGG patients treated with IMRT, regardless of the additional presence of the 1p/19q codeletion (Table 1) [38]. OS was not affected, and the PFS data will also need to be confirmed through further, more extensive investigations.

It has been suggested that the efficacy of novel treatments could exploit specific tumor mutational profiles (Table 1) [39,40]. For instance, relapsed HGG often shows higher mutational loads than the primary tumor. Part of this additional mutational load carries the characteristic mutations induced by the drug TMZ in non-coding sequences of tumor cells. These mutated sequences can be attacked with CRISPR/CAS, which causes fragmentation of the genome of these cells and their elimination (Table 1; Figure S3) [41]. This could therefore be a new treatment approach aimed at eliminating hypermutant cells, which often present characteristics of greater malignancy. Furthermore, particular mutational profiles and gene signatures may help to predict the response of the tumor to RT (Table 1) [42,43].

An increasingly popular and perhaps promising strategy is to combine RT and drug treatment with specific diets that can increase their effectiveness (pharmaconutrition) [82,83]. Even in this case, large randomized, double-blind clinical trials will be necessary to reach any conclusions of sufficient solidity.

*Radiation-Induced Damage*

**Figure 4:**
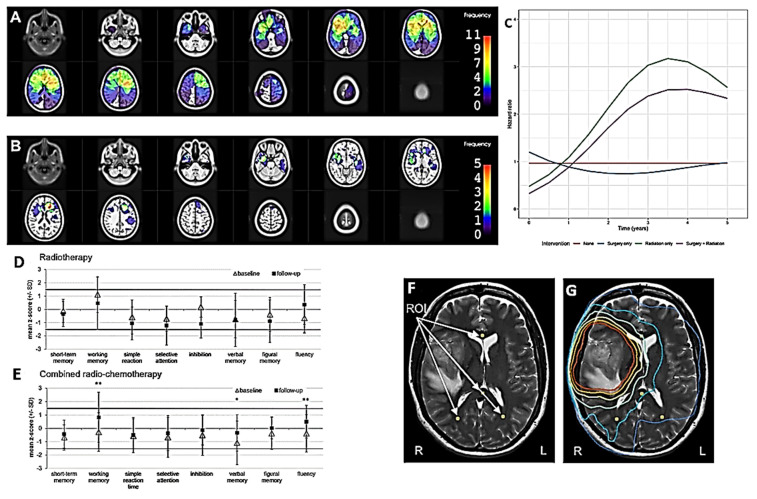
Clinical studies. Advances in understanding and treatment of RID in adult HGG patients. (**A**,**B**) Heatmaps for planning target volumes (PTVs) and radiation-induced damage (RID) for the HGG subgroup. The color bar shows the frequency of occurrence as the number of contours in that location. (**A**) PTVs of all RT courses in the area of RID are superimposed. (**B**) Frequency of occurrence of RID (contrast-enhancing lesion) (after [44], with permission). (**C**) Time-dependent hazard ratio plot for intervention types (no treatment “none” was used as the reference group) (after [47], with permission). (**D**) Mean cognitive performance (in z-scores) and standard deviations (error bars) in NeuroCog FX subtests at baseline and follow-up (median 7.1 years [range 4.6–11.0] after baseline) for patients treated with RT only (n = 7) or (**E**) with combined radio-chemotherapy (n = 29). Statistically significant changes in cognitive performance between baseline and follow-up are indicated by asterisks (* *p* < 0.05, ** *p* < 0.01) (after [50], with permission). (**F**,**G**) Synthetic T2-weighted image with region of interest (ROI) and dose distribution. In (**F**), there is an example of ROI placements in a synthetic T2-weighted image of a patient. ROIs are placed in the lower part of the parietal lobe (**left** and **right**), in the genu and splenium of the corpus callosum. In (**G**), the same T2-weighted image is displayed, but with an overlay of the isodose lines of 10, 20, 30, 40, 50, and 57 Gy (after [51], with permission).

Radiation-induced damage (RID) may not be radiologically distinguishable from tumor progression and may require histopathological analysis. It may consist of irreversible tissue necrosis or pseudoprogression. An analysis of cases of radiation damage in patients with HGG undergoing RT has been performed (Table 1; Figure 4A,B) [44]. This type of damage is understandably more frequently localized in proximity to the residual irradiated lesion. The onset of tissue damage is dependent on the intensity of the dose and its spatial distribution. Despite the low doses used, tissue damage may nevertheless occur even after hyperfractionated RT. There are several brain areas that are particularly susceptible to damage by RT. The subventricular zones (SVZ), rich in stem cells, may be among them. Steroids may be useful in reducing symptoms of cerebral radionecrosis, but their long-term use may induce serious side effects.

In addition to necrosis, although rarer, RT can induce secondary HGG even after more than ten years. Treatment is not standardized, partly because of the rarity of these cases and partly because they are difficult to treat due to the risk of inducing further radiation damage, which reduces the life expectancy of the patient. Generally, an attempt is made to treat them with a combination of RT and CT, sometimes based on the molecular characteristics of the secondary tumor (Table 1) [45]. Bugdadi et al. reported a rare case of radiation-induced glioma (RIG) with epithelioid features and the presence of molecular features consistent with RIG (Table 1) [46]. The patient had been treated with craniofacial brachytherapy 70 years earlier, and such a late onset would be a unique case. The induction of GB by brachytherapy was attributed to the biomolecular features of the tumor, compatible with a radiation-induced tumor. The growth rate of this tumor was very slow, and despite not having completed the adjuvant CT cycle after surgery, no recurrence was observed during the following 5 years. Subjecting this case to multiple histopathological reviews to gather a consensus about the real histopathological nature of the tumor might be appropriate.

Stroke is a well-established complication in cancer patients, the risk of which is particularly high in patients with HGG. This risk may be increased after RT treatments. The extent of this increase and its timing are poorly studied. Kouli and colleagues investigated the relationship between RT and stroke-specific mortality in patients with primary brain tumors (Table 1; Figure 4C) [47]. The risk of stroke is relatively low in patients with primary HGG, and this risk may increase due to treatment. Two patterns of mortality have been observed: lower mortality due to acute stroke in untreated patients and higher mortality due to delayed risk following RT and CT treatment. The latter would peak 3.5–4 years after diagnosis.

An additional side effect of RT may consist of neurocognitive decline in the patient. Actually, we do not know much about the risk factors that may lead to this eventuality. It has been supposed that it is a complex set of factors related to the tumor, the patient, and the treatment that are difficult to decipher (Table 1; Figure S4) [48]. Among them, recurrent tumor growth is most likely to play a significant role. Less important, however, seems to be the negative effect of CT with temozolomide. This alkylating agent has the great advantage of limited toxicity coupled with relative efficacy, and this also applies to its relative lack of negative effects on HGG patients (Table 1) [49]. Pertz et al. conducted a prospective study to evaluate the possible harmful effects of hippocampal irradiation (Table 1; Figure 4E,F) [50]. In this study, neurocognitive damage was assessed to be less than expected even for total doses greater than 50 Gy. Quantitative MRI (qMRI), unlike conventional MRI, has the advantage of highlighting even small variations in the structure of specific tissues such as white matter after RT and CT, for example in relation to the concentration of myelin (Table 1; Figure 4G,H) [51]. Mental suffering and cognitive decline following a diagnosis of HGG require adequate and timely psychological and psychotherapeutic care. Fischl and colleagues studied whether the effectiveness of such care was reduced due to the distance from the oncology center of HGG patients residing within a large rural area (Table S1) [84]. No particular relevance of the spatial distance between the place of residence and the location of the oncology center was found regarding the adequacy of psychotherapeutic care.

#### 3.1.2. Pediatric HGG

Compared to the section on adult patients, this pediatric section is relatively short and based heavily on case reports and small series. Fortunately, this is largely attributable to the much lower incidence of HGG in pediatric patients compared to adults. For instance, GB represents 48.6% of all CNS tumors in adults, where it peaks in the fifth/sixth decade of age [85]. Its incidence is more than tenfold lower (4.1%) in the age group 0–14 years, where CNS tumors are predominantly low-grade [85]. Based on this hierarchy of evidence, it should be emphasized that most data reported in this section only allow for suggestions for further larger studies rather than established conclusions.

Xie et al. studied the therapeutic effect of DDR inhibition by AZD1390 in combination with radiotherapy (RT) (Table 1) [52]. The cytotoxic effect of RT was enhanced in the presence of AZD1390, both in vitro and in vivo orthotopic models. Contrary to previous studies, the therapeutic effect did not vary in the presence or absence of p53 mutations. The suggested mechanism involved not only the inhibition of ATM but also the involvement of ATR, which would cause the induction after RT of mutagenic and toxic damage due to the welding of non-homologous DNA sequences and a consequent increase in genomic instability. Clinical trials of AZD1390 + RT in pediatric patients with HGG have been proposed.

Sharma M. et al. identified a small molecule, MTX-241F, that inhibits members of the EGFR and PI3 kinase family, including the radiation damage repair protein DNA-PK (Table 1) [53]. MTX-241F appears to readily cross the BBB, reaching micromolar concentrations in the mouse brain. Since RT causes double-strand breaks that are repaired by homologous recombination (HR) and non-homologous DNA end-joining (NHEJ), pediatric mouse HGG were treated with a combination of an ATM inhibitor, which reduces NHEJ, and MTX-241F, which would inhibit HR after RT. Synthetic lethality was observed under these conditions, suggesting this pharmacological combination should be explored in a clinical setting.

**Figure 5:**
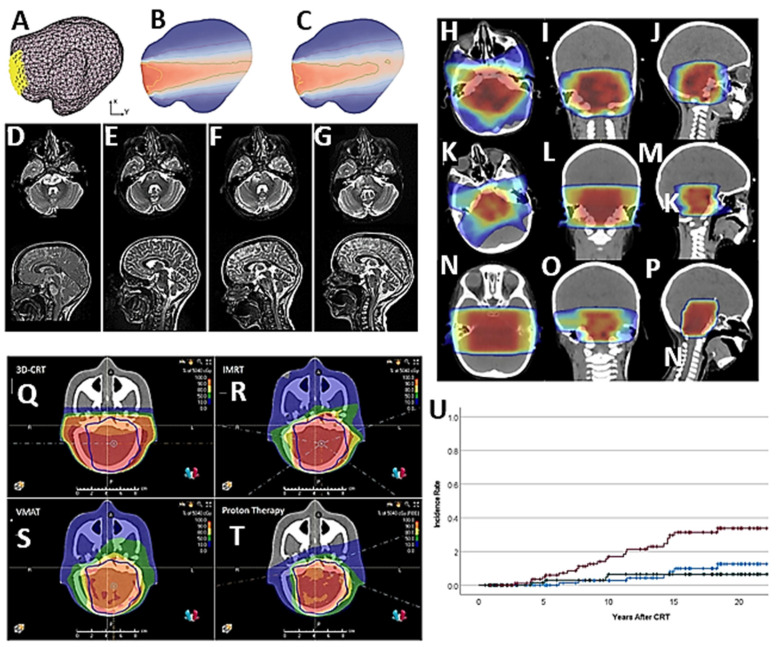
Clinical studies. Advances in RT for pediatric HGG patients. (**A**–**C**) Dose distribution in real geometries of DIPG tumors irradiated from a single patch. (**A**) Meshed geometry with an initial patch shown in yellow; (**B**) dose distribution considering homogeneous DIPG tumor properties; (**C**) dose distribution considering heterogeneous DIPG tumor properties (after [54], with permission). (**D**–**G**) Imaging of the tumor in T2w-MRI axial and sagittal at the following timepoints: (**D**) initial diagnosis 01/2020; (**E**) 6 months after the initial irradiation, (**F**) progression 01/2021; (**G**) 1 month after the second re-irradiation (after [55], with permission). (**H**–**P**) First irradiation isodose distribution in axial (**H**), coronal (**I**), and sagittal (**J**): 20 Gy (dark blue), 32 Gy (light blue), 40 Gy (green), 45 Gy (yellow), 48 Gy (orange), and 54 Gy (red). Second irradiation isodose distribution in axial (**K**), coronal (**L**), and sagittal (**M**): 7.5 Gy (dark blue), 12 Gy (light blue), 15 Gy (green), 17.5 Gy (yellow), 19 Gy (orange), and 22 Gy (red). Third irradiation isodose distribution in axial (**N**), coronal (**O**), and sagittal (**P**): 4 Gy (dark blue), 8 Gy (light blue), 9 Gy (green), 10 Gy (yellow), 11 Gy (orange), and 12 Gy (red) (after [56], with permission). 3D-CRT (**Q**), IMRT (**R**), VMAT (**S**), and PBS proton therapy (**T**) plans showing isodoses and PTV (blue) as computed by the treatment planning system (after [57], with permission). (**U**) Incidence rate for the development of any neoplasm (upper curve, red), meningioma (middle curve, blue), or HGG (lower, green) after conformal photon RT for ependymoma, age < 3 years (after [58], with permission).

The calculation of the radiation dose to be applied to the tumors is of fundamental importance to obtain maximum therapeutic efficacy. It is increasingly evident that this calculation should take into account the existence within the tumor of cellular populations with different degrees of radiosensitivity. This is especially true for pediatric HGG, which often presents characteristics of marked heterogeneity. Juma et al. presented a simplified model for the determination of photonic efficacy in diffuse intrinsic pontine glioma (DIPG) and neuroblastoma tumors to illustrate the importance of cellular heterogeneity in the calculation of the RT dose to be applied (Table 1; Figure 5A–C) [54]. Concomitant chemotherapy (CT)/radiotherapy (RT) followed by repeated reirradiation with dose de-escalation was used by Bergengruen and coworkers in the treatment of a pediatric patient with DIPG (Table 1; Figure 5D–G) [55]. The patient, with good performance status, tolerated this type of treatment well. The actual benefit in terms of increased OS and PFS, as well as reduction in neurocognitive side effects, is, however, difficult to quantify, given the asymptomatic nature of the patient and the study, which was conducted on a single case. Lo Greco and collaborators studied the impact of cycles of re-irradiation in young patients affected by DIPG (Table 1; Figure 5H–P) [56]. All patients had been treated with vinorelbine and nimotuzumab and 54 Gy of RT. At later stages of progression, the ethics committee decided to subject the young patients to clinical and instrumental checks to investigate the possibility of further cycles of RT. Two to three additional cycles of RT showed efficacy in slowing tumor progression as determined by clinical neurological evaluation and radiological evaluation by MRI with contrast. One of the three cases examined survived 23 months. The authors suggested that although our current knowledge is not sufficient to explain such clinical responses, two cycles of re-irradiation could be beneficial in some selected patients.

De Saint-Hubert et al. developed and validated a Monte Carlo framework to evaluate the exposure of healthy tissues to radiation, causing various types of pathologies in children (Table 1; Figure 5Q–T) [57]. They concluded that proton therapy is the type of RT which, with equal efficacy, is the most capable of sparing adverse events in neighboring healthy tissues.

Conformal photon RT promised to spare normal tissue and was introduced more than 25 years ago to improve outcomes for these vulnerable patients. Long-term results for those first treated with conformal methods provide valuable information on radiation-induced tumors and serve as a comparison against newer methods (Table 1; Figure 5U) [58].

There is a lack of guidelines for the re-irradiation of pediatric HGG. To fill this gap, the Swedish Workgroup of Pediatric RT has developed guidelines for the re-irradiation of pediatric DIPG, ependymoma, germinoma, and medulloblastoma. These have been in clinical practice since 2019 in all pediatric RT centers in Sweden and are updated annually based on accumulated clinical experience. These national guidelines were presented by Embring et al. (Table 1) [59]. Gordon and colleagues described the combined use of 5-aminolevulinic acid (5-ALA) and caesium-131 (^131^Cs) GammaTile therapy in a pediatric patient with recurrent HGG (Table 1) [60]. 5-ALA was used to better define the surgical resection of the tumor and the target volume of RT. GammaTile is a brachytherapy treatment specifically designed for use inside the brain: the neurosurgeon places GammaTile(s) precisely where and when treatment will help the most, at the tumor site immediately after tumor removal. In this study, the RT was performed with Gamma Tile with ^131^Cs, delivering an optimized beam to the tumoral residue. The treatment was suggested by the authors to be more effective than a classic external photonic RT treatment with better localization and sparing of the organs and tissues at risk.

Williamson et al. studied the possible effect of varying the timing of RT application and dosage on pediatric HGG (Table 1) [61]. They concluded that advancing RT within 90 days of diagnosis or increasing the radiation dosage did not benefit the young patients.

RT can temporarily slow the tumor’s growth but is not curative. Mueller and colleagues studied the safety, tolerability, and distribution of MTX110 (aqueous panobinostat) administered by convective administration (CED) in patients with newly diagnosed DIPG who completed RT [86]. Repeated CED of MTX110 with real-time imaging with gadoteridol was tolerable for irradiated patients with DIPG. The median OS of 26.1 months was prolonged compared to the survival medians of “historical” patients with DIPG.

Cerebral edema is a frequent adverse event after RT. Alsahlawi and colleagues described a beneficial effect of BV treatment in a 14-year-old patient who required prolonged treatment with steroids (Table 1) [62]. BV allowed the suspension of dexamethasone treatment and its side effects, while still reducing cerebral edema, although not the tumor volume.

Rhodes et al. performed prospective neuropsychological assessments (two weeks after RT and six months later) in children diagnosed with CNS malignancies, including a large sample of patients with DIPG (Table 1) [63]. Results were reported as cross-sectional population and longitudinal individual patient characteristics. Before RT, children with DIPG showed significant deficits in attention, performance, and tests of processing speed and verbal learning/memory. Younger children demonstrated worse parent-reported behavioral regulation, depression, and social withdrawal than older children. Six months after RT, older children showed poorer socialization than younger children. Longitudinally, children with DIPG showed short-term improvements immediately after RT on all tests, but these improvements were short-lived and almost completely lost six months later.

RT treatment of childhood gliomas seeks to reduce as much as possible the exposure of normal tissues to limit neurocognitive decline in the years to come. Especially with grade II–III tumors, it is not uncommon for young patients to reach adulthood. Most of these patients acquire relative functional independence [87]. A minority, however, due to cognitive decline that occurs after the end of treatments and the chronicity of the disease, are unable to acquire independence and autonomy.

As aforementioned for adults, there is a risk that secondary tumors may arise after RT for children as well, especially meningiomas, gliomas, and cavernomas. This risk, although limited, must be communicated [88]. In general, the efficacy of therapies on these secondary tumors is not dissimilar to that observed with primary tumors of the same type.

## 4. Conclusions

At the population level, standard treatments for HGG have seen few changes in the past two decades, with novel treatment options and survival improvements confined to a few, often poorly characterized, patients. The road to progress in radiotherapy (RT), leading to an improved median OS of HGG patients, is still long and winding [89]. While the present review may be limited by the relatively short publication period, HGG research remains potentially impacted, especially at the clinical level, by issues with trial design, such as treating patients with advanced disease progression only, using “historical” data as controls, and repurposing drugs developed for completely different purposes than intended. Addressing these aspects of HGG research might improve its efficacy, which often remains low despite the associated costs.

In the second half of 2023, several important advances have been made in both the adult and pediatric settings, under various RT aspects including treatment planning, innovative fractionation, tumor radiosensitization, and the development and use of radioisotopes and radiopharmaceuticals. Looking at the two most significant examples, IORT with relatively safe RT doses may represent an approach that should be deeply investigated, with the potential to improve clinical outcomes [32,90]. Despite the high rate of complications and treatment failures, multiple studies on the use of IORT in the treatment of HGG have reported survival results comparable to or even superior to conventional methods [91]. As highlighted in this article for other therapeutic strategies, the lack of adequately controlled randomized trials, as well as the small sample sizes analyzed to date, warrants further prospective randomized controlled studies to confirm the data collected and better identify the specific patient populations that may benefit most from IORT. Notably, a randomized RP3 study (NCT02685605) is currently ongoing to provide a deeper understanding of the benefits and drawbacks of using IORT in the treatment of HGG.

Ultra-hyperfractionated radiotherapy (UHFRT) emerges as a promising protocol to improve survival rates of HGG patients, in part by reshaping the tumor microenvironment and enhancing the antitumor immune response [75,76,92]. The low toxicity of UHFRT may allow for its combination with other therapies and facilitate the treatment of multiple infiltrating lesions [93,94]. In this case, the first preclinical evidence of efficacy in the treatment of HGG dates back decades. However, this research has suffered a prolonged stalemate due to the inability to reproduce in vitro results in inadequate animal models that poorly mimic the development of human tumors. Only recently have orthotopic animal tumors induced by glioma-initiating cells (GICs) allowed for the therapeutic action of UHFRT to be studied in vivo [95]. This now paves the way for adequately controlled and randomized clinical trials, which alone can provide a definitive answer regarding the therapeutic potential of UHFRT for HGG.

In conclusion, in the search for new effective therapies for HGG, two critical aspects of clinical trial design should be reconsidered. First, adequate patient randomization and internal controls should be implemented in Phase II clinical trials. In this regard, reliance on comparisons with “historical” data may produce misleading results that may lead to the implementation of costly and unsuccessful phase III clinical trials [96,97]. Second, research over the past twenty years has taught us that it is extremely difficult to treat effectively HGG that is already progressing [98]. The efficacy of new therapies should be studied in patients with minimal residual disease—that is, in patients who have recently completed the conventional Stupp therapy.

## Figures and Tables

**Table 1:** Advances in radiotherapy (RT) for high-grade gliomas (HGG).

*Adult HGG*		
		
Major finding	Experimental system	Ref.
		
Preclinical studies		
Concomitant irradiation and TRAM-34 treatment is efficacious in a preclinical glioma mouse model.	Syngeneic, immune-competent orthotopic SMA-560/VM/Dk glioma mouse model.	[9]
IWR1 treatment effectively radiosensitizers glioma stem cells and helps to overcome the survival advantages promoted by LDR.	C6 glioma cell line.	[10]
Inhibiting SOAT1 enhances the efficacy of RT in gliomas, both in vitro and in vivo, by promoting sensitivity to ferroptosis. *	U87 and U251 cells. Mouse orthotopic glioblastoma (GB) models transfected with U87 cells.	[11]
A combination of unbiased genetic screens and CRISPR-Cas9-based gene therapy is feasible for identifying potential synthetic lethal genes and, by extension, therapeutic targets. *	Human GB cell line LN229. Patient-derived GB cell lines (GBM02) and glioma stem cells (GSC05). Orthotopic mouse xenografts.	[12]
Nanoparticles absorbed by glioma cells can produce a significant radiosensitizing effect, probably due to the action of secondary electrons generated by the magnetite core, whereas the dextran shell of the nanoparticles used in these experiments appears to be rather stable under radiation exposure.	A172 and Gl-Tr glioma cells.	[13]
Long intergenic non-coding (LINC)00839, modified by METTL3-mediated m6A, exerts tumor progression and radiation resistance by activating Wnt/β-catenin signaling. *	Two glioma initiating cells (GICs) (MES28 and GSC2907) and two neural stem cells (NSCs) (HNP1 and NESA). Thirty primary GB specimens and twenty recurrent GB specimens.	[14]
Ataxia telangiectasia and Rad 3-related kinase (ATR) expression is elevated in the tumor margin regions of human GB samples and is associated with poorer survival. ATR promotes GB cell invasion via the internalization and trafficking of integrins. Pharmacological inhibition of ATR reduces GB invasion. *	Primary GB cell lines E2, G7, R15, Ox5 and S24 were derived from patients’ resected tumors.	[15]
Proton therapy offers some advantages over other modern conformal photon-based therapies when used in combination with H_2_ for the treatment of central nervous system (CNS) malignancies.	Authors’ opinion	[16]
Interventions designed to target regulators of glucose metabolism, such as PKM2, rather than specific HGG metabolic pathways, have the potential to improve the RT outcomes in GB patients. *	Primary human GB lines, GBM217, GBM374, and GBM382. Patient-derived xenografts (GBM38).	[17]
Silencing of *REV3L* and *REV7* inhibited radiation resistance via inactivating the PI3K/AKT/mTOR pathway, suggesting that targeting DNA polymerase ζ may be a new strategy to reduce the RT resistance of glioma.	Glioma tissues (n = 40) and para-cancerous normal tissues (n = 40) from patients with glioma.	[18]
Radio dynamic therapy with 5-aminolevulinic acid (5-ALA) could suppress colony formation under normal and hypoxic conditions in lymphoma cells.	A human Burkitt lymphoma cell line (Raji) and two human brain lymphoma cell lines (HKBML and TK).	[19]
		
Clinical studies		
*RT treatment planning*		
SRT is a viable treatment modality with a significant survival contribution. Since this may have a favorable prognostic effect on survival in patients with tumor size < 2 cm, early diagnosis of recurrence and a decision to re-irradiate a smaller tumor during follow-up were recommended.	A total of 59 patients with 64 HGG lesions were re-irradiated in a single center with the CyberKnife Robotic Radiosurgery System.	[20]
Although the use of immobilization equipment may decrease the image quality in the RT setup, it does not affect organ delineation, and the image quality is still satisfactory for this purpose. Also, the use of immobilization equipment in the RT setup increased registration accuracy.	Computed tomography (CTOM) and magnetic resonance imaging (MRI) images from 11 patients with HGG, all of whom were immobilized with a thermoplastic mask and headrest.	[21]
T1w-MRI DL-AC could segment all brain organs at risk (OARs) except the lacrimal glands, which cannot be easily visualized on T1w-MRI. Editing contours on MRI before model training improved geometric performance. MRI DL-AC in RT may improve consistency, quality and efficiency, but requires careful editing of training contours.	Retrospective glioma cases were randomly selected for training (n = 32, 47) and validation (n = 9, 10) for MRI and CTOM, respectively. Clinical contours were edited using an international consensus (gold standard) based on MRI and CTOM.	[22]
The presence of OARs in addition to planned target volume (PTV) does not provide new knowledge for the network, and only by defining the PTV and its location in the imaging slices does the dose distribution become predictable. Therefore, the only-PTV method, by eliminating the process of introducing OARs, can reduce the overall time of treatment design by IMRT in patients with glioma tumors.	The data of 99 patients with glioma tumors referred for IMRT treatment were used, with 90 patients’ images serving as the training dataset and the others as the test dataset.	[23]
In terms of dose calculation and patient positioning accuracy, the studied MRI-only method demonstrated its clinical feasibility for RT planning of the brain.	Clinical validation of dose calculation accuracy was performed by a retrospective evaluation for 25 glioma and 25 brain metastasis patients. Patient positioning verification accuracy of sCTOM images was retrospectively evaluated for 10 glioma and 10 brain metastasis patients based on clinical cone-beam-computed tomography (CBCTOM) imaging.	[24]
The simulated tumor cell invasion is a stronger predictor of overall survival than the segmented gross tumor volume (GTV), indicating the importance of using mathematical models for cell invasion to assist in the definition of the target for HGG patients.	A model describing tumor infiltration into normal tissue was applied to 93 HGG cases. Tumor infiltration maps and corresponding isocontours with different cell densities were produced. ROC curves were used to seek correlations between the patient’s overall survival (OS) and the volume encompassed by a particular isocontour.	[25]
After identification of a significant correlation between computed growth parameters and clinical and biological data, the potential of tumor growth modelling for individualized therapy of GB was highlighted.	124 patients from The Cancer Genome Atlas (TCGA) and 397 patients from the UCSF Glioma Dataset were assessed for significant correlations between clinical data, genetic pathway activation maps (generated with PARADIGM; TCGA only), and infiltration (Dw) as well as proliferation (ρ) parameters stemming from a Fisher-Kolmogorov growth model	[26]
STRIDeR pathway plans achieved a similar robustness to manual pathways with improved PTV doses. Geometric and linear quadratic model uncertainties can be incorporated into the STRIDeR pathway to facilitate robust optimization.	For ten HGG reRT patient cases, uncertainties were applied and cumulative doses resumed.	[27]
A four-gene RT-related signature can predict the radiation efficacy of WHO grade 4 glioma patients, and may provide a reference for clinical treatment options.	Human glioma cell lines U87MG and U251MG. A total of 282 patients included in the TCGA database, and 265 patients in the Chinese glioma genome atlas (CGGA) database. Eighty-three WHO grade 4 glioma patients who received RT in the authors’ center.	[28]
		
*Innovative fractionation*		
Dose-escalated hypoRT in 20 fractions produced survival outcomes outperforming “historical” data for frail patients.	Forty frail HGG patients who were treated with hypoRT were retrospectively analyzed.	[29]
Short-course palliative hypofractionated RT in patients with poor-prognosis HGG is associated with stable and/or improved QoL scores in several domains, making it a viable resource-sparing regimen.	Forty-nine patients with poor-prognosis HGG were accrued for a prospective study of short-course palliative hypofractionated RT (35 Gy/10 fractions/2 weeks).	[30]
For recurrent GB, re-RT with SRT/FSRT plus regorafenib is a safe treatment.	Twenty-one patients with a histological or radiological diagnosis of recurrent GB who received re-RT by SRS/FSRT and regorafenib as second-line systemic therapy were evaluated	[31]
Surgery for brain metastases with intraoperative RT (IORT) in the posterior fossa (PF) is safe and feasible.	A retrospective analysis of nine patients (five female) receiving surgery for brain metastases and undergoing IORT at a single institution.	[32]
		
*Radioisotopes and radiopharmaceuticals*		
Optical imaging and nuclear imaging can work complementarily with multimodal imaging in the design and evaluation of anticancer nanomedicine, offering an MIL-101(Fe)/PEG-FA-based pharmaceutical with potential in tumor endoRT.	Fluorescence imaging (FI) was combined with nuclear imaging to systematically evaluate the tumor inhibition of new nanomedicines from living cancer cells to the whole body.	[33]
		
*Radiosensitization*		
Differential alterations in proteomic expression pre- vs. post-completion of concurrent chemotherapy (CT)/radiotherapy (RT) are present with the addition of VA. Using pre- vs. post-data, prognostic proteins emerged in the analysis.	A total of 29 patients received CT/RT plus VA, and 53 patients received CT/RT alone.	[34]
Temozolomide (TMZ) combined with IMRT could improve the level of serum factor in postoperative glioma patients, strengthen the immune function of the patients, and effectively facilitate the clinical comprehensive efficacy without increasing adverse reactions.	A total of 124 patients with HGG were selected and randomly divided into the study group and the control group, with 62 cases in each group.	[35]
Bevacizumab (BV) can be an option for heavily pretreated patients with rGII-III glioma with contrast enhancement. In this study, BV displayed activity in a subgroup of patients.	In this retrospective study, eighty-one adult patients with histologically proved rGII-III glioma were included.	[36]
The prognostic value of O6-methylguanine-DNA methyltransferase (MGMT) promoter methylation in patients with HGG was confirmed. Sequencing of whole promoter CpG islands demonstrated that methylation of particular CpG sites might predict clinical outcomes more precisely.	A total of 95 consecutive HGG patients treated with surgical gross total resection, followed by concomitant RT/CT and adjuvant CT, were included in this retrospective observational study	[37]
This retrospective study showed that the 1-year progression-free survival (PFS) of patients with isocitrate-dehydrogenase (*IDH)*-mutated was better than *IDH*-wild-type patients. Patients with *IDH*-mut/*MGMT*-methylation had the best prognosis for PFS in the whole subgroup. There was no difference for OS.	Forty-five patients with treated grade II-IV glioma	[38]
While multiple host, tumor, and drug-related features may limit the delivery and efficacy of targeted therapies for patients with HGG, genotype-matched targeted therapies confer favorable clinical outcomes.	The distribution of hotspot mutations in 388 GBs from the TCGA was evaluated. Mutations were matched with 54 targeted therapies, followed by a comprehensive evaluation of the drug’s biochemical properties and its clinical efficacy in HGG. The clinical outcomes of a cohort of patients with HGG harboring targetable mutations, reviewed at the Johns Hopkins MTB, were finally assessed.	[39]
To validate molecularly matched targeted therapies in glioma patients, the prevalence and persistence of actionable molecular alterations in patient tissue must be considered.	Forty-one glioma patients, of whom thirty-two had isocitrate dehydrogenase (IDH) wild-type GB.	[40]
CRISPR-mediated targeting of highly repetitive loci enables rapid elimination of GB cells, an approach that was termed “genome shredding.” Importantly, in the patient’s recurrent GB, unique repeat sequences with TMZ mutational signature were identified, and their CRISPR targeting enabled cancer-specific cell ablation.	The mutational landscape progression in a patient’s primary and recurrent GB was determined, and *Cas9*-targetable repeat elements were uncovered.	[41]
A new nine-gene radiosensitivity-related prognostic risk signature that can predict the prognosis of patients with glioma who received RT was established. The nomogram showed potential to predict the prognosis of patients with glioma treated using RT.	Radio-sensitive and radio-resistant glioma cell lines (M059J and M059K). A total of 169 GB samples and 5 normal samples from the TCGA database, as well as 80 GB samples and 4 normal samples from the GSE7696 set.	[42]
The high expression of TMEM59L might enhance RT sensitivity by increasing ROS-induced DNA damage and inhibiting the DNA damage repair process.	The differentially expressed genes were screened based on RNA sequencing in 15 pairs of primary and recurrent GBs that underwent RT.	[43]
		
*Radiation-induced damage (RID)*		
Accelerated hyperfractionated RT can lead to RID despite computationally low EQD2_2_ and biologically effective dose (BED_2_) in HGG patients. The anatomical location of RID corresponded to the general tumor distribution of gliomas and metastases. The SVZ might be a particularly vulnerable area.	Thirty-three histopathologically confirmed cases of RID after photon-based RT for primary or secondary CNS malignancies were included.	[44]
RT and CT may improve the survival of patients with RIGs. Furthermore, molecular features may influence the clinical, locoregional, and pathological features of RIG.	Two patients, a 32-year-old man and a 50-year-old man, developed GB more than 20 years after RT monotherapy for germinoma with or without mature teratoma.	[45]
Despite not receiving the full course of adjuvant CT after surgery and RT, the patient displayed no signs of recurrence during a 5-year follow-up. This RIG should be further studied to reveal potential unique clinical and molecular characteristics, as well as to better predict survival and treatment response.	Description of a rare case of most likely radiation-induced glioma (RIG) with epithelioid features and the presence of molecular features consistent with RIG.	[46]
The risk of stroke specific mortality (SSM) is low in patients with primary brain tumors and is not increased by RT. Two different patterns were observed: acute stroke mortality in patients receiving no treatment, and delayed stroke mortality in patients receiving RT (+/− surgery), with the latter peaking 3.5–4 years after diagnosis.	A total of 85,284 patients with primary brain tumor diagnoses were analyzed.	[47]
Decline in neurocognitive functions in HGG patients is multifactorial and can be attributed to an amalgam of various tumor-, patient-, and treatment-related factors.	Fifty-three newly diagnosed HGG patients who underwent maximal safe resection.	[48]
TMZ proved to be a secure treatment with no negative side effects on cognition or level of daily autonomy, even at the highest dosage used.	Sixty-one HGG patients.	[49]
Multimodal glioma therapy seems to affect neurocognition less than generally assumed. Even patients with unilateral hippocampal irradiation with >50 Gy showed no profound cognitive decline in this series.	Seventy-one glioma patients (WHO grade 1–4) were serially evaluated with neurocognitive testing and quality of life questionnaires.	[50]
In the long-term follow-up for glioma patients, qMRI is a powerful tool for detecting small changes, such as a decrease in myelin concentration, in normal-appearing white matter (NAWM) after RT.	NAWM was analyzed in 10 patients across 83 MR examinations performed before and after surgery and after RT.	[51]
		
*Pediatric HGG*		
		
Major finding	Experimental system	Ref
AZD1390 potentiated radiation in pHGG cells representing distinct molecular subgroups. AZD1390, combined with radiation, increased survival in *TP53* wild-type and *TP53* mutant xenografts. ATR inhibition was synthetically lethal to pHGG cells resistant to the combination. *	SU-diffuse intrinsic pontine glioma (DIPG)XIII cells. SJ-DIPG7 and SJ-DIPG37 orthotopic xenografts.	[52]
Proof-of-concept evidence to support advanced development of MTX-241F for the treatment of DIPG.	SU-DIPG-13P * (hereafter called DIPG 13P *) cells; HSJD-DIPG 007 (hereafter called DIPG 007) cells, and SU-DIPG-XIII (hereafter called DIPG-XIII) cells. Human SF7761 and SF8628 cell lines. Flank xenograft model with luciferase DIPG 13P * cells. Orthotopic xenograft model with luciferase DIPG 13P * cells.	[53]
Cellularity data extracted from DIPG were utilized to demonstrate the importance of heterogeneity in RT dose calculation.	The finite element method (FEM) was employed to simulate the photon flux and dose deposition in real cases of DIPG tumors.	[54]
A second course of re-irradiation can be an additional tool in patients with progressive disease after first- and second-line irradiation. It is unclear whether and to what extent this contributes to progression-free survival prolongation, and if—since the examined patient was asymptomatic—progression-associated neurological deficits can be alleviated.	Retrospective case report of a 6-year-old boy with DIPG receiving a second course of re-irradiation (with 21.6 Gy) as part of an individual multimodal approach in a patient with very low symptom burden.	[55]
Even though it goes beyond the understanding of conventional radiobiology, first and second re-RT in pediatric patients with progressive DIPG may represent a feasible and safe approach, capable of increasing overall survival and disease-free survival in selected patients and improving their quality of life.	A retrospective case series on three children with progressive DIPG.	[56]
Proton therapy allows for a significant reduction in the out-of-field doses and secondary cancer risk in selected organs.	Organ doses were calculated for treatment of a diffuse midline glioma (DMG) (50.4 Gy with 1.8 Gy per fraction) on a 5-year-old anthropomorphic phantom with 3D-conformal RT (3D-CRT), IMRT, volumetric-modulated arc therapy (VMAT), and intensity-modulated pencil beam scanning (PBS) proton therapy.	[57]
Long-term results of children treated using photon conformal RT after surgery demonstrate that adjuvant RT resulted in long-term disease control and functional independence.	A total of 101 patients < 3.1-years-old old were treated with conformal and intensity modulated photon therapy after definitive surgery for intracranial ependymoma.	[58]
This article presents the Swedish national guidelines on re-irradiation in pediatric CNS tumors.	The Swedish Workgroup of Paediatric RT (SBRTG) updated the national guidelines on re-irradiation in pediatric CNS tumors (DIPG, ependymoma, germinoma and medulloblastoma).	[59]
The utilization of 5-ALA and GammaTile therapy yielded clinically superior tumor debulking and effective RT dose localization, sparing organs at risk, respectively.	GammaTile caesium^131^ brachytherapy with 5-aminolevulinic acid Fluorescence-guided resection.	[60]
There was no benefit to early RT timing when RT was initiated within 90 days of diagnosis or a higher RT dose was used in this dataset.	A total of 498 pHGG patients were included. The median age was 15 years (range, 0–21), common diagnoses were astrocytoma (55%) and GB (30%), 73.5% underwent surgical resection, and 90.2% received CT.	[61]
The use of bevacizumab despite the absence of clear evidence of radionecrosis allowed for a significant decrease in the amount of brain edema.	A 14-year-old boy with a steroid-dependent refractory tumor whose longstanding dexamethasone treatment was successfully discontinued after a course of bevacizumab.	[62]
Children with DIPG exhibited short-term improvements immediately post-radiation in performance-based attention tests and parent-reported behavior, including attention, hyperactivity, behavioral regulation, and executive function. However, these improvements did not persist, and a significant decline was documented in tests of attention by six months. Clinical implications for professionals working with children with DIPG and recommendations for cognitive remediation and quality-of-life interventions were provided.	Children diagnosed with CNS malignancies, including 21 children with DIPG.	[63]

* Possible clinical trial-worthy strategy.

## Data Availability

No new data were created or analyzed in this study. Data sharing does not apply to this article.

## Supplementary Materials

The following supporting information can be downloaded at: https://www.mdpi.com/article/10.3390/ijms27020662/s1. References [99,100] are cited in the Supplementary Material.

## Abbreviations

5-ALA, 5-aminolevulinic acid; AA, anaplastic astrocytoma; Ac, actinium; ATR, ataxia telangiectasia kinase Rad-related protein; BBB, blood–brain barrier; BED, biologically effective dose; BNCT, boron neutron capture therapy; BV, bevacizumab; CE, contrast enhanced; CED, convection enhanced delivery; CGGA, Chinese glioma genome atlas; CI, confidence interval; CNS, central nervous system; CT, chemotherapy; CTOM, computed tomography; CTV, clinical target volume; DIPG, diffuse intrinsic pontine glioma; DMG, diffuse midline glioma; FLAIR, fluid attenuated inversion recovery; GB, glioblastoma; GIC, glioma initiating cell; GTV, gross tumor volume; HGG, high-grade glioma; IDH, isocitrate dehydrogenase; IORT, intraoperative RT; KPS, Karnofsky performance status; LINC, long intergenic non coding; MGMT, O6-methylguanine-DNA methyltransferase; MRI, magnetic resonance imaging; OAR, organs at risk; OS, overall survival; PFS, progression free survival; PTV, planned target volume; RID, radiation-induced damage; ROI, region of interest; ROS, reactive oxygen species; RT, radiotherapy; TCGA, the cancer genome atlas; TMZ, temozolomide; UHFRT, ultra-hyperfractionated RT.
