# Supplementary material for: Radiotherapy for High-Grade Gliomas in Adults and Children: A Systematic Review of Advances Published in the Second Half of 2023
Source: Int J Mol Sci. 2026 Jan 9;27(2):662. doi: 10.3390/ijms27020662 (PMC12840602; doi:10.3390/ijms27020662)
Supplement: Supplementary file 1 [file ijms-27-00662-s001.zip › ijms-4060563- Supplementary materials.pdf]

Supplementary materials

**Table S1. Advances in RT for HGG**		
**Adult patients**		
Major finding	Experimental system	Ref
Preclinical studies		
In an orthotopic rat glioma model using F98 cells treated with boron neutron capture therapy (BNCT), the boron carrier 4-iodophenylbutanamide (BC-IP), improves animal survival to a lesser extent in comparison to 4-borono-L-phenylalanine (L-BPA).	In vitro rat glioma cell lines F98 and C6. In vivo orthotopic rat glioma model using F98 cells.	[80]
Clinical studies		
Innovative fractionation		
Border zone (BZ)-SRS with BV was feasible and well-tolerated. There is no significant survival benefit using BZ-SRS with BV compared to institutional “historical” controls.	Sixteen patients with histologically confirmed GB with recurrent disease who had received prior first-line treatment with fractionated RT and CT and were eligible for SRS, were enrolled.	[71]
Radiosensitization		
The addition of BEV to TMZ in this patient group neither improves nor negatively impacts quality of life.	155 WHO grade 2 and 3 glioma patients	[81]
Radiation-induced damage		
In studies involving novel therapeutic agents, robust and properly timed dosimetric patterns of failure analysis may help evaluate the biologic aspects of these agents.	The pattern of failure of 109 newly diagnosed GB patients per the 2016 WHO classification who received conformal RT with concomitant and adjuvant TMZ was reviewed.	[99]
Butterfly GB has a poor prognosis. Unless a patient is planned for adjuvant CT/RT following biopsy, they should be managed conservatively. This avoids unnecessary procedural interventions, along with their associated morbidities and costs.	80 cases of bGB were identified. These patients were managed with biopsy ± adjuvant therapy (36), with RT alone without biopsy (3), or with surgical resection (3). Thirty-eight cases of suspected bGB were managed conservatively: receiving no oncological treatment, biopsy or tumour resection.	[100]
The distance of IDHwt glioma patients’ homes from their cancer center, even in a rural area, does not significantly influence the rate of psycho-oncological care.	229 adult malignant isocitrate dehydrogenase wild-type (IDHwt) glioma patients of this retrospective cohort fulfilled the inclusion criteria for analysis.	[84]

Fig. S1 PRISMA 2020 for Abstracts Checklist.

PRISMA

PRISMA 2020 for Abstracts Checklist

Section and Topic	Item #	Checklist item	Reported (Yes/No)
**TITLE**			Page
Title	1	Identify the report as a systematic review.	1 of 27
**BACKGROUND**			
Objectives	2	Provide an explicit statement of the main objective(s) or question(s) the review addresses.	1 of 27
**METHODS**			
Eligibility criteria	3	Specify the inclusion and exclusion criteria for the review.	1 of 27
Information sources	4	Specify the information sources (e.g. databases, registers) used to identify studies and the date when each was last searched.	1 of 27
Risk of bias	5	Specify the methods used to assess risk of bias in the included studies.	1 of 27
Synthesis of results	6	Specify the methods used to present and synthesise results.	1 of 27
**RESULTS**			
Included studies	7	Give the total number of included studies and participants and summarise relevant characteristics of studies.	1 of 27
Synthesis of results	8	Present results for main outcomes, preferably indicating the number of included studies and participants for each. If meta-analysis was done, report the summary estimate and confidence/credible interval. If comparing groups, indicate the direction of the effect (i.e. which group is favoured).	1 of 27
**DISCUSSION**			
Limitations of evidence	9	Provide a brief summary of the limitations of the evidence included in the review (e.g. study risk of bias, inconsistency and imprecision).	1 of 27
Interpretation	10	Provide a general interpretation of the results and important implications.	1 of 27
**OTHER**			
Funding	11	Specify the primary source of funding for the review.	1 of 27
Registration	12	Provide the register name and registration number.	1 of 27

Fig. S2 PRISMA 2020 Checklist.

PRISMA

PRISMA 2020 Checklist

Section and Topic	Item #	Checklist item	Location where item is reported
**TITLE**			Pages
Title	1	Identify the report as a systematic review.	1 of 27
**ABSTRACT**			
Abstract	2	See the PRISMA 2020 for Abstracts checklist.	1 of 27
**INTRODUCTION**			
Rationale	3	Describe the rationale for the review in the context of existing knowledge.	2 of 27
Objectives	4	Provide an explicit statement of the objective(s) or question(s) the review addresses.	2 of 27
**METHODS**			
Eligibility criteria	5	Specify the inclusion and exclusion criteria for the review and how studies were grouped for the syntheses.	2-3 of 27
Information sources	6	Specify all databases, registers, websites, organisations, reference lists and other sources searched or consulted to identify studies. Specify the date when each source was last searched or consulted.	2-3 of 27
Search strategy	7	Present the full search strategies for all databases, registers and websites, including any filters and limits used.	2-3 of 27
Selection process	8	Specify the methods used to decide whether a study met the inclusion criteria of the review, including how many reviewers screened each record and each report retrieved, whether they worked independently, and if applicable, details of automation tools used in the process.	2-3 of 27
Data collection process	9	Specify the methods used to collect data from reports, including how many reviewers collected data from each report, whether they worked independently, any processes for obtaining or confirming data from study investigators, and if applicable, details of automation tools used in the process.	2-3 of 27
Data items	10a	List and define all outcomes for which data were sought. Specify whether all results that were compatible with each outcome domain in each study were sought (e.g. for all measures, time points, analyses), and if not, the methods used to decide which results to collect.	2-3 of 27
	10b	List and define all other variables for which data were sought (e.g. participant and intervention characteristics, funding sources). Describe any assumptions made about any missing or unclear information.	2-3 of 27 Tables & Figs
Study risk of bias assessment	11	Specify the methods used to assess risk of bias in the included studies, including details of the tool(s) used, how many reviewers assessed each study and whether they worked independently, and if applicable, details of automation tools used in the process.	2-3 of 27
Effect measures	12	Specify for each outcome the effect measure(s) (e.g. risk ratio, mean difference) used in the synthesis or presentation of results.	2-3 of 27
Synthesis methods	13a	Describe the processes used to decide which studies were eligible for each synthesis (e.g. tabulating the study intervention characteristics and comparing against the planned groups for each synthesis (item #5)).	2-3 of 27
	13b	Describe any methods required to prepare the data for presentation or synthesis, such as handling of missing summary statistics, or data conversions.	2-3 of 27
	13c	Describe any methods used to tabulate or visually display results of individual studies and syntheses.	2-3 of 27
	13d	Describe any methods used to synthesize results and provide a rationale for the choice(s). If meta-analysis was performed, describe the model(s), method(s) to identify the presence and extent of statistical heterogeneity, and software package(s) used.	2-3 of 27
	13e	Describe any methods used to explore possible causes of heterogeneity among study results (e.g. subgroup analysis, meta-regression).	2-3 of 27
	13f	Describe any sensitivity analyses conducted to assess robustness of the synthesized results.	2-3 of 27
Reporting bias assessment	14	Describe any methods used to assess risk of bias due to missing results in a synthesis (arising from reporting biases).	n.a.
Certainty assessment	15	Describe any methods used to assess certainty (or confidence) in the body of evidence for an outcome.	2-3 of 27

PRISMA

PRISMA 2020 Checklist

Section and Topic	Item #	Checklist item	Location where item is reported
**RESULTS**			Pages
Study selection	16a	Describe the results of the search and selection process, from the number of records identified in the search to the number of studies included in the review, ideally using a flow diagram.	4-21 of 27
	16b	Cite studies that might appear to meet the inclusion criteria, but which were excluded, and explain why they were excluded.	n.a.
Study characteristics	17	Cite each included study and present its characteristics.	4-21 of 27 Tables and Figs
Risk of bias in studies	18	Present assessments of risk of bias for each included study.	n.a.
Results of individual studies	19	For all outcomes, present, for each study: (a) summary statistics for each group (where appropriate) and (b) an effect estimate and its precision (e.g. confidence/credible interval), ideally using structured tables or plots.	4-21 of 27 Tables and Figs
Results of syntheses	20a	For each synthesis, briefly summarise the characteristics and risk of bias among contributing studies.	Tab. 1
	20b	Present results of all statistical syntheses conducted. If meta-analysis was done, present for each the summary estimate and its precision (e.g. confidence/credible interval) and measures of statistical heterogeneity. If comparing groups, describe the direction of the effect.	4-21 of 27 Tables and Figs
	20c	Present results of all investigations of possible causes of heterogeneity among study results.	4-21 of 27
	20d	Present results of all sensitivity analyses conducted to assess the robustness of the synthesized results.	Tables and Figs
Reporting biases	21	Present assessments of risk of bias due to missing results (arising from reporting biases) for each synthesis assessed.	n.a.
Certainty of evidence	22	Present assessments of certainty (or confidence) in the body of evidence for each outcome assessed.	4-21 of 27 Tables and Figs
**DISCUSSION**			
Discussion	23a	Provide a general interpretation of the results in the context of other evidence.	21-22 of 27
	23b	Discuss any limitations of the evidence included in the review.	21-22 of 27
	23c	Discuss any limitations of the review processes used.	21-22 of 27
	23d	Discuss implications of the results for practice, policy, and future research.	21-22 of 27
**OTHER INFORMATION**			
Registration and protocol	24a	Provide registration information for the review, including register name and registration number, or state that the review was not registered.	22 of 27
	24b	Indicate where the review protocol can be accessed, or state that a protocol was not prepared.	22 of 27
	24c	Describe and explain any amendments to information provided at registration or in the protocol.	22 of 27
Support	25	Describe sources of financial or non-financial support for the review, and the role of the funders or sponsors in the review.	22 of 27
Competing interests	26	Declare any competing interests of review authors.	22 of 27
Availability of data, code and other materials	27	Report which of the following are publicly available and where they can be found: template data collection forms; data extracted from included studies; data used for all analyses; analytic code; any other materials used in the review.	22 of 27

n.a., not applicable.

From: Page MJ, McKenzie JE, Bossuyt PM, Boutron I, Hoffmann TC, Mulrow CD, et al. The PRISMA 2020 statement: an updated guideline for reporting systematic reviews. BMJ 2021;372:n71. doi: 10.1136/bmj.n71. This work is licensed under CC BY 4.0. To view a copy of this license, visit https://creativecommons.org/licenses/by/4.0/

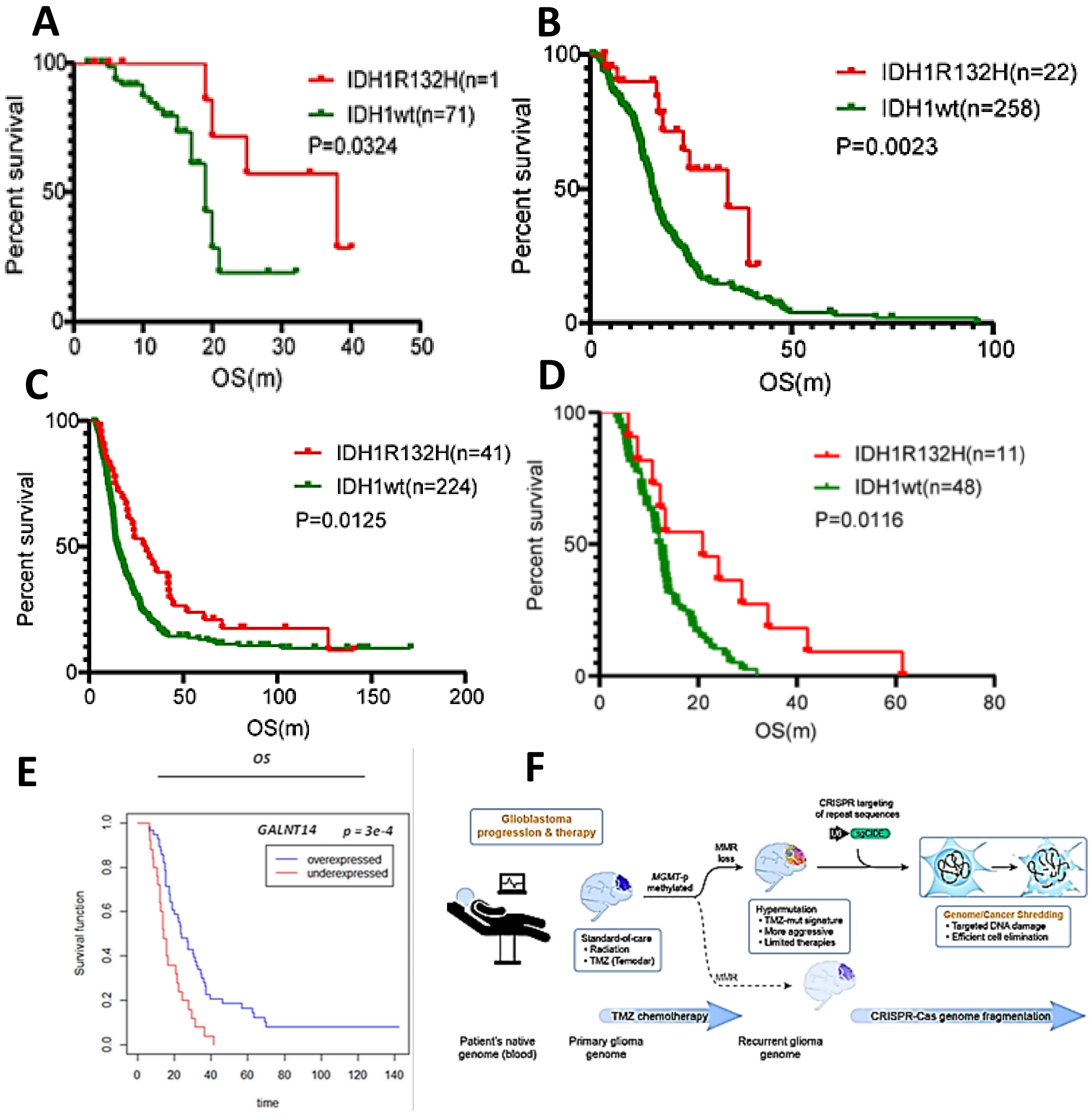

Figure S3. (**A-F**) Survival curves of WHO Grade 4 glioma patients with different IDH1 gene status. (**A**) Differences in OS in WHO Grade 4 glioma patients receiving RT at the authors' centre. (**B**) Survival analysis of patients in the TCGA database. All patients received RT. OS of WHO grade 4 IDH-mutant astrocytoma patients was better than that of IDH1 wild-type. (**C**) Survival analysis of patients in the CGGA database. All patients received RT. The prognosis of WHO grade 4 IDH-mutant astrocytoma patients was better than that of IDH1 wild-type. (**D**) Stratified analysis of patients in the CGGA database. Among the patients receiving RT alone, the prognosis of WHO grade 4 IDH-mutant astrocytoma patients was better (after [28] with permission). (**E**) Kaplan–Meier curves for OS by difference in GALNT14 expression pre- vs. post-completion of chemoirradiation (after [34] with permission). (**F**) Schematic of GB progression with frontline therapy including radiation, TMZ CT, and surgical resection. Highlighted is CRISPR-Cas "genome/cancer shredding," a strategy for recurrent tumours that induces DNA damage and cell elimination through targeting of repetitive sequences in the genome (after [41] with permission).

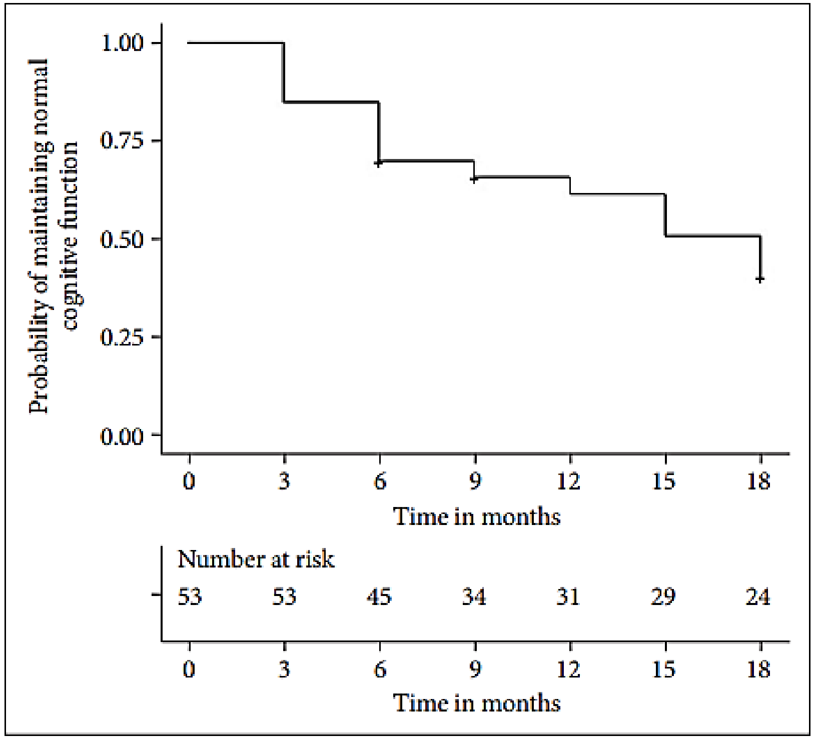

Figure S4. (D) Probability of maintaining normal cognitive function at different points in time for the study population (after [48] with permission).

Supplementary abbreviations

3D-CRT, 3D-conformal RT; BC-IP, boron-conjugated 4-iodophenylbutanamide; BZ, border zone; CBCTOM, cone-beam computed tomography; CCeCTOM, clinical contours edited based on CTOM anatomy; CRT, conformal RT; Cs, caesium; CSF, cerebrospinal fluid; CT/RT, chemoirradiation; CTu, CT unedited autosegmentation DL, deep learning; DSC, dynamic susceptibility contrast; DTI, diffusion tensor tractography; ENT, ear, nose and throat; EQD2, equivalent dose in 2 Gy fractions; ESTRO-EORTC, european society for radiotherapy and oncology and european organisation for research and treatment of cancer; EVO, evofosfamide; FSRT, fractionated stereotactic RT; GKSR, gamma knife radiosurgery; GLI, glioma associated oncogene; H^+, proton; H_2, hydrogen; HH, hedgehog; HR, homologous recombination; IDL, isodose line; IF, immunofluorescence; IMRT, intensity modulated RT; IWR1, inhibitor of Wnt1; L-BPA, 4-borono-L-phenylalanine; LET, linear energy transfer; LGG, low-grade glioma;, long non coding; Lu, lutetium; MGMT-p, MGMT promoter; MMR, mismatch repair; MTB, molecular tumor board; NAWM, normal appearing of white matter; NF 1, neurofibromatosis type 1; NHEJ, non-homologous end joining; NT, normal tissue; ODN, anti-sense oligonucleotide; PBS, pencil beam scanning; PF, posterior fossa; PP2Ac, catalytic subunit of protein phosphatase-2A; PRV, planning organ at risk volume; qMRI, quantitative MRI; reRT, re-irradiation; RIG, radiation induced glioma; rvSIB-IMRT, small-volume intensity-modulated RT with simultaneous integrated boost; sCTOM, deep-learning based synthetic computed tomography; SRS, stereotactic radiosurgery; SRT, stereotactic RT; SSM, stroke specific mortality; STRIDeR, support tool for re-irradiation decisions guided by radiobiology; SVZ, subventricular zone; TGM, tumour growth model; UCSF, University of California at San Francisco; VA, valproic acid; VM, vasculogenic mimicry; VMAT, volumetric modulated arc therapy; WHO, World Health Organization.
